# MiR92b-3p synthetic analogue impairs zebrafish embryonic development, leading to ocular defects, decreased movement and hatching rate, and increased mortality

**DOI:** 10.1007/s13353-022-00732-w

**Published:** 2022-10-24

**Authors:** Kilian Kranert, Maciej Woźny, Piotr Podlasz, Krzysztof Wąsowicz, Paweł Brzuzan

**Affiliations:** 1 Department of Environmental Biotechnology, Institute of Engineering and Environment Protection, Faculty of Geoengineering, University of Warmia and Mazury in Olsztyn, ul. Słoneczna 45G, 10-709 Olsztyn, Poland; 2 Department of Pathophysiology, Forensic Veterinary Medicine and Administration, Faculty of Veterinary Medicine, University of Warmia and Mazury in Olsztyn, ul. Oczapowskiego 13, 10-718 Olsztyn, Poland

**Keywords:** Embryogenesis, Gene expression regulation, In vivo transfection, Microinjections, MiR92b, Ocular development

## Abstract

The aim of this study was to examine the effect of microRNA 92b-3p (MiR92b-3p) overexpression on the embryonic development of zebrafish. A synthetic MiR92b-3p analogue (mirVana™ mimic, in vivo-ready) was injected at doses up to 5 ng/embryo into the yolk sac of embryos (2–16 cell stage). At 24 h post fertilization (hpf), the locomotor activity of the embryos was measured, and after hatching (72 hpf), the rates of malformation occurrence, hatching, and mortality were determined. Next, the larvae were fixed for histological and molecular examinations. Exposure to the MiR92b-3p mimic impaired embryonic development, leading to increased occurrence of malformations (i.e., pericardial edema, spine curvature, smaller eyes), decreased locomotor activity and hatching rate, and increased mortality. Importantly, the mimic affected retinal differentiation and lens formation during zebrafish embryogenesis, which suggests that MiR92b-3p could be an important factor in the regulation of fish embryogenesis and ocular development. The expression level of MiR92b-3p was substantially higher in the exposed larvae than in the untreated larvae, indicating that the mimic was successfully delivered to the zebrafish. Although screening of potential MiR92b-3p target genes suggested some changes in their expression levels, these results were inconclusive. Together, this study indicates that MiR92b-3p mimic impairs zebrafish embryonic development, and further research is necessary to identify the MiR92b-3p–regulated cell pathways involved in the impairment of the fish’s development.

**Supplementary Information:**

The online version contains supplementary material available at 10.1007/s13353-022-00732-w.

## Introduction

MicroRNAs (miRNAs) are non-coding RNA molecules that are approximately 22 nucleotides long and are highly conserved in multicellular organisms (Ahkin Chin Tai and Freeman 2020). These small RNAs are expressed in strict spatiotemporal patterns during different stages of embryonic development; some are tissue specific, while others occur ubiquitously in many tissues (Wienholds et al. 2005). Predominantly due to a part of their sequence known as the seed region, miRNAs can recognize and bind to specific sequences in the 3′ untranslated region of mRNA, targeting those transcripts for degradation or repressing their translation to proteins (Ha and Kim 2014). Thus, miRNAs are important regulators of gene expression and play important roles in cell specification and function (DeVeale et al. 2021). Dysregulation of miRNA expression not only can disrupt normal animal development but also may play a role in some diseases (Matsuyama and Suzuki 2019).

MiR92b-3p belongs to the *miR25* gene family, which can be found in a range of organisms, including flies, fish, and humans. It is involved in numerous biological processes and the development of critical diseases (miRBase gene family: MIPF0000013; Kozomara et al. 2019). For example, MIR92b-3p acts as a tumor suppressor by targeting specific pro-oncogenic pathways, as shown in human pancreatic cancer tissues and cell lines (Long et al. 2017). Furthermore, altered tissue expression of MIR92b-3p plays a role in tumor progression and is predictive of survival of prostate cancer patients (Wang et al. 2021). Despite recent progress in knowledge of the role of *MIR92b* in human malignancies, much less progress has been made on its role in the embryonic development of vertebrates, including fish.

In adult zebrafish, *mir92b* is expressed in many tissues in various organs; however, in embryos, this miRNA has only been found in certain regions of the developing brain (Kloosterman et al. 2006). In zebrafish and Japanese flounder, expression of two members of the *mir92* family disrupts early embryonic development (Li et al. 2011, 2020). For example, although injections of zebrafish embryos with a mixture of MiR92a and MiR92b drastically inhibited endodermal cell formation at the blastula and gastrula developmental stages, inhibition of MiR92 in the developing embryos resulted in aberrant left–right body patterning (Li et al. 2011). Importantly, the developmental abnormalities in fish embryos have been attributed to MiR92 control of *gata5* and *sox17* expression (Li et al. 2011, 2020), transcription factors involved in regulating embryonic development and determining endodermal cell fate in vertebrates (Reiter et al. 2001; She and Yang 2015). Additionally, it has been suggested that this miRNA regulates other genes that are important for early embryonic development (Li et al. 2011).

In this study, the aim was to examine the effect of MiR92b-3p overexpression on zebrafish embryonic development. Towards this aim, a synthetic MiR92b-3p analogue (mirVana™ mimic, in vivo-ready) was injected at doses of 0.5 or 5 ng/embryo into the yolk sac of embryos at an early stage of development. To assess the response of the zebrafish to this treatment, the locomotor activity of the embryos was measured at 24 h post fertilization (hpf), and the rates of malformation occurrence, hatching, and mortality among the larvae were determined after hatching (72 hpf). To provide a more detailed description of the exposed embryos’ morphology, the histological structure of 72 hpf zebrafish sections was examined using histochemical staining and light microscopy. Finally, to gain insight into the possible regulatory mechanisms of MiR92b-3p, the mRNA expression levels of several target genes, selected on the basis of literature reports, were screened. The results of this study indicate that MiR92b-3p is likely involved in zebrafish ocular development.

## Material and methods

### Zebrafish spawning

Mature zebrafish (wild type, Tübingen strain) were housed in the facility at the Faculty of Veterinary Medicine, University of Warmia and Mazury (27.0 ± 0.2 °C, salinity 700–800 µS/cm). The fish were kept with a photoperiod of 14 h light/10 h darkness and fed three times daily: twice with dry food (GEMMA Micro Zebrafish; Skretting) and once with *Artemia* sp. *nauplii* (HE > 230 000 NPG; Ocean Nutrition TM). To obtain embryos, two male and three female zebrafish were set in a breeding tank the night prior to spawning with a separation sheet between the sexes. In the morning, the separation sheets were removed to allow spawning, then the embryos were collected, washed with E3 medium (5 mM NaCl, 0.17 mM KCl, 0.33 mM CaCl2, 0.33 mM MgSO_4_), transferred to sterile petri dishes filled with E3, and stored in an incubator (28.5 °C).

### Fish exposure

For the zebrafish microinjections, a high-quality purified synthetic analogue of mature MiR92b-3p (#MIMAT0003218; mirVana™ mimic, in vivo-ready; Thermo Fisher Scientific) was purchased. Injection solutions consisted of MiR92b-3p mimic diluted in phosphate buffer saline, mixed with phenol red to track injection accuracy. For embryo exposure, intra-yolk microinjections were carried out with selected embryos at the 2–16 cell stages using an InjectMan NI2 manipulator and a FemtoJet injector (Eppendorf) with 5 nL volumes of mimic concentrations of 0.5 ng or 5 ng/embryo (MIM 0.5 ng and MIM 5 ng groups). Additionally, to track the delivery of the mimic injected into the yolk, a separate batch of embryos was injected with a dose of 1 ng/embryo of a synthetic MiR92b-3p mimic that was 3′-labeled with Alexa Fluor 555 (AF-MIM group). Untreated embryos served as a negative control group (control). Additional embryos injected with a mirVana™ *Caenorhabditis elegans* MiR39-3p mimic (#MIMAT0000010; Thermo Fisher) at a dose of 5 ng/embryo served as an additional group (mismatch; MIS) to check the effects of the microinjections and the vehicle solvent. To our best knowledge, mature miRNA sequence of *C. elegans* MiR39-3p does not show any significant homology to any known chordate miRNA sequences (miRBase). Embryos from each group were incubated separately at 28.5 °C in E3 solution, which was exchanged daily.

### Mortality, malformation, and hatching rate

Mortality, malformation, and hatching rate were assessed based on a previously described protocol (Majewski et al. 2018). Briefly, the percentages of mortality and of malformed and hatched embryos and larvae were determined under a SteREO Discovery.V8 microscope (Zeiss) at 24-h intervals over the 3 days following fertilization. Mortality was identified by a missing heartbeat, coagulation of the embryos, failure to develop somites, or presence of a non-detached tail. Zebrafish embryos were scored for developmental abnormalities including lack of pigmentation; heart edema and changes in heart size (> 50%); malformations of the head, tail, and heart; scoliosis; yolk deformities; and growth retardation. For calculating the larval hatch rate, hatching was considered successful when a larva’s head or tail broke out of the chorion, and the rate was expressed as the percentage of living embryos that had hatched during a given sampling time.

### Movement analysis

Locomotor behavioral measurements (burst count, burst duration) were performed with 24 hpf embryos. Video clips were recorded using a SteREO Discovery.V8 microscope and a DLT-Cam PRO 6.3-MP camera (Delta Optical). The clips were analyzed with DanioScope software (Noldus).

### Histopathological analysis

At the end of the experiment, 72 hpf zebrafish larvae were fixed in Davidson’s fixative at room temperature. The fixed samples were dehydrated in a series of ethanol dilutions, cleared with xylene, and embedded in paraffin with an automatic tissue processor (Leica 1020). Paraffin blocks were prepared with a Leica EG1150C tissue embedder. Next, 4.5-µm paraffin sections were cut with a Leica RM2255 rotary microtome, and the sections were put on chrome alum-gelatin-coated slides. Sections were deparaffinized and stained in an automatic stainer (Leica Autostainer XL) with Mayer’s hematoxylin and alcohol eosin. Sections were dehydrated with alcohol, cleared with xylene, embedded with Entellan, and coverslipped. Sections were viewed with a Nikon Eclopse 80i brightfield microscope and photographed using a CCD camera and NIS-Elements Basic Research software (Nikon).

### Total RNA isolation and cDNA synthesis

At 72 hpf, zebrafish larvae (*n* = 25 per replicate) were transferred to 1.5-mL tubes, then the E3 solution was aspired, and the samples were stored at − 80 °C until extraction. Total RNA was extracted with a Total RNA mini kit (A&A Biotechnology) followed by incubation with TURBO™ DNAse (Thermo Fisher Scientific) at 37 °C for 30 min. After genomic DNA digestion, the RNA samples were cleaned up using a PureLink RNA mini kit (Invitrogen). The quantity and integrity of the total RNA samples were evaluated with an Agilent Bioanalyzer 2100 using an Agilent RNA 6000 Nano kit (Agilent Technologies). In these samples, RNA integrity was acceptable (RIN ~ 8.0).

For the screening the mRNA targets, reverse transcription (RT) was performed using a RevertAid™ First Strand cDNA synthesis kit (Thermo Scientific). The reaction mixture contained 1 μg of total RNA and 5 μM of oligo(dT)_18_ primer. The samples were incubated at 65 °C for 5 min, then chilled on ice, and the following components were added: 4 μL of 5 × Reaction Buffer, 20 U of RiboLock™ RNase Inhibitor, 1 mM of dNTP mix, and 200 U of RevertAid™ M-MuLV Reverse Transcriptase. The reaction was carried out at 42 °C for 60 min, then terminated by heating at 70 °C for 5 min. Synthesized cDNA samples were diluted (2 ×) and stored at − 80 °C until amplification.

To profile MiR92b expression, a protocol based on polyadenylated RNA and stem-loop RT was used (Biggar et al. 2014; Florczyk et al. 2019). Polyadenylation of miRNA was performed using a Poly(A) Polymerase Tailing kit (Lucigen). The reactions contained 1 μL of 10 × polyadenylate polymerase buffer, 1 μL of adenosine triphosphate (ATP, 10 mM), 0.5 μL of *Escherichia coli* poly(A)polymerase (4 U), 1 μg of total RNA, and RNase-free water for a final volume of 10 μL. The reaction mixtures were incubated at 37 °C for 30 min, followed by 95 °C for 5 min to terminate the reaction, then transferred to ice. Next, RT was performed as described above for mRNA target analysis, with one modification: instead of using oligo(dT)_18_ primer, an aliquot of 10 μL polyadenylated RNA from the previous step was incubated with 1 μL of 100 μM universal stem-loop RT primer (5′-CTC ACA GTA CGT TGG TAT CCT TGT GAT GTT CGA TGC CAT ATT GTA CTG TGA GTT TTT TTT TVN-3′). Synthesized cDNA samples were diluted (20 ×) and stored at − 80 °C until amplification.

### Selection of target and reference genes

To gain insight into the possible regulatory mechanisms of MiR92b-3p, several target genes were selected based on the existing literature and their mRNA expression levels were screened: (i) *gata5* and *sox17* were screened because these genes interact with MiR92 (Li et al. 2011, 2020); (ii) *pax6a* and *pax6b* were selected because these genes are involved in the development and function of the eye (Takamiya et al. 2015). For expression normalization of the mRNA targets, *actb1*, *eef1a1l1*, and *ubc* were selected and validated as possible reference genes (Macaulay et al. 2016; Leach et al. 2021). For details of the primers for the potential targets and reference genes, see Supplement 1.

### qPCR

This analysis was performed on a Quant Studio 5 instrument (Applied Biosystems). Each reaction consisted of 5 μL of 2X Power SYBR Green Master Mix (Applied Biosystems), an optimized concentration of primers (Supplement 1), 1 µL of the diluted cDNA, and PCR-grade water for a final volume of 10 μL. The reaction was carried out in duplicate as follows: 95 °C for 10 min, then 40 cycles of 95 °C for 15 s and 60 °C for 1 min. To check the quality of the PCR products, a dissociation analysis was performed after each run. PCR product homogeneity was confirmed with agarose gel electrophoresis (Supplement 1).

For selecting the most stable reference gene for normalization of target mRNA expression, geNorm was used (Vandesompele et al. 2002), which indicated that *ubc* was the best candidate (Supplement 1). The miRNA levels were normalized with rnu6 (Zhuang et al. 2014) as the reference gene because it showed acceptable expression variance across all investigated samples (Supplement 1). Relative expression was calculated according to Livak and Schmittgen (2001).

### Statistical analysis

The relative expression values of the miRNA and mRNA targets were log-transformed prior to further analysis (Limpert et al. 2001; Limpert and Stahel 2011). Then, differences between the exposed and control groups were assessed using one-way ANOVA followed by Tukey’s HSD test. These calculations were performed with SPSS Statistics 27 (IBM; USA).

To assess how the treatment affected the probabilities of zebrafish mortality, hatching, and malformation occurrence, logistic regression was used to calculate *p* values for differences between the control and treatment groups using base R, version 3.5.3 (R Core Team 2019), and the emmeans package (Lenth 2019).

## Results

### Mortality, hatching, and occurrence of malformations

**Fig. 1:**
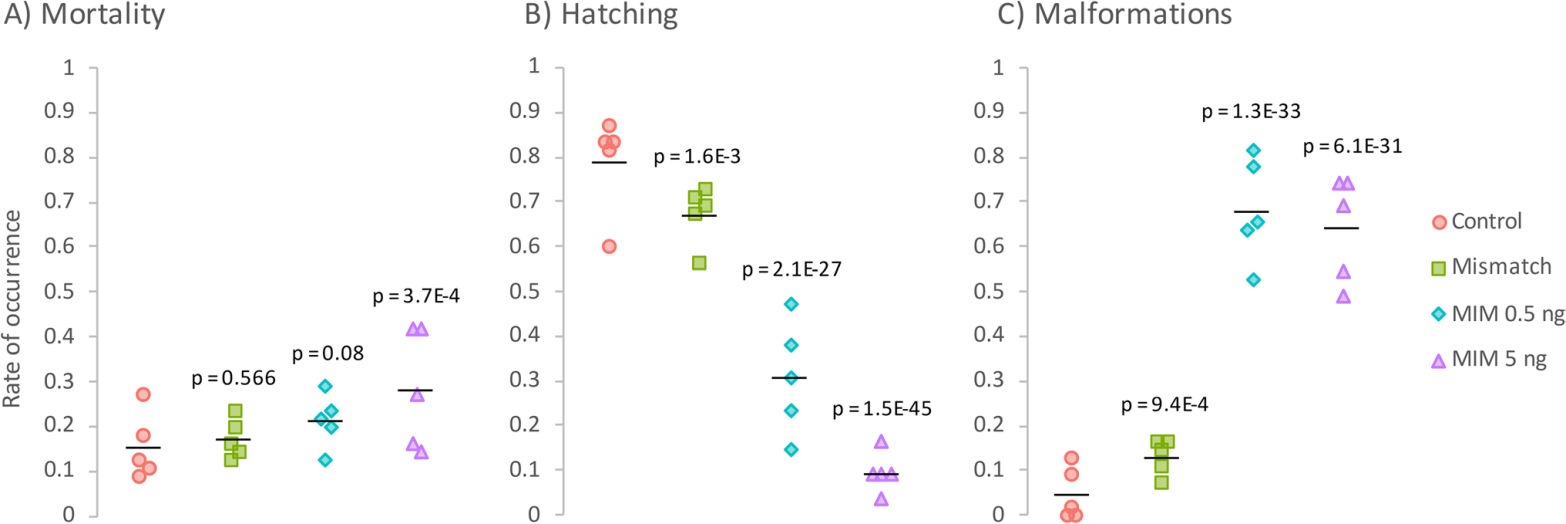
**A** Mortality, **B** hatching, and **C** malformations in zebrafish larvae 72 h after intra-yolk microinjections of MiR92b-3p mimic at 0.5 ng/embryo (MIM 0.5 ng; cyan diamonds) or 5 ng/embryo (MIM 5 ng; violet triangles), or intra-yolk microinjections with *C. elegans* MiR39-3p mimic at 5 ng/embryo (mismatch; green squares) compared to untreated zebrafish larvae (control; red circles). Individual points show rate of occurrence in individual Petri dishes used for incubation (i.e., replicates; *n* = 5 per group). Black horizontal line indicates mean rate of occurrence. *p* values for the difference in the rate of occurrence between the control and exposed groups were calculated using logistic regression

At 72 hpf, mortality was almost twice as high in the MIM 5 ng group as in the untreated (control) group (28.4% vs. 15.6%), and this difference was statistically significant (Fig. 1A; *p* < 0.001). Although the differences between the control group and both the MIM 0.5 ng group and the MIS group were not statistically conclusive (*p* > 0.05), mortality was somewhat higher in both of these groups (17.5% and 21.5%, respectively) than in the control group.

Although MIS-exposure decreased the hatching rate of the zebrafish embryos, MIM-exposure decreased their hatching rate to a much larger extent (Fig. 1B). Compared to the control group, the probability of hatching was 12% lower in the MIS group, 48.4% lower in the MIM 0.5 ng group, and 69.8% lower in the MIM 5 ng group. All these differences were statistically significant (*p* < 0.002).

Similarly, MIS-exposure increased the frequency of malformations, but MIM-exposure increased it to a much greater extent (Fig. 1C). The percentage of malformations was 8.4% higher in the MIS group than in the control group. In contrast, it was 63.6% higher in the MIM 0.5 ng group than in the control group. Interestingly, the higher dose of MIM (5 ng/embryo) did not appear to further increase the percentage of malformations, as the percentage was 59.6% higher in this group than in the control group. All the differences relative to the control group were statistically significant (*p* < 0.001).

### Gross description of malformations

**Fig. 2:**
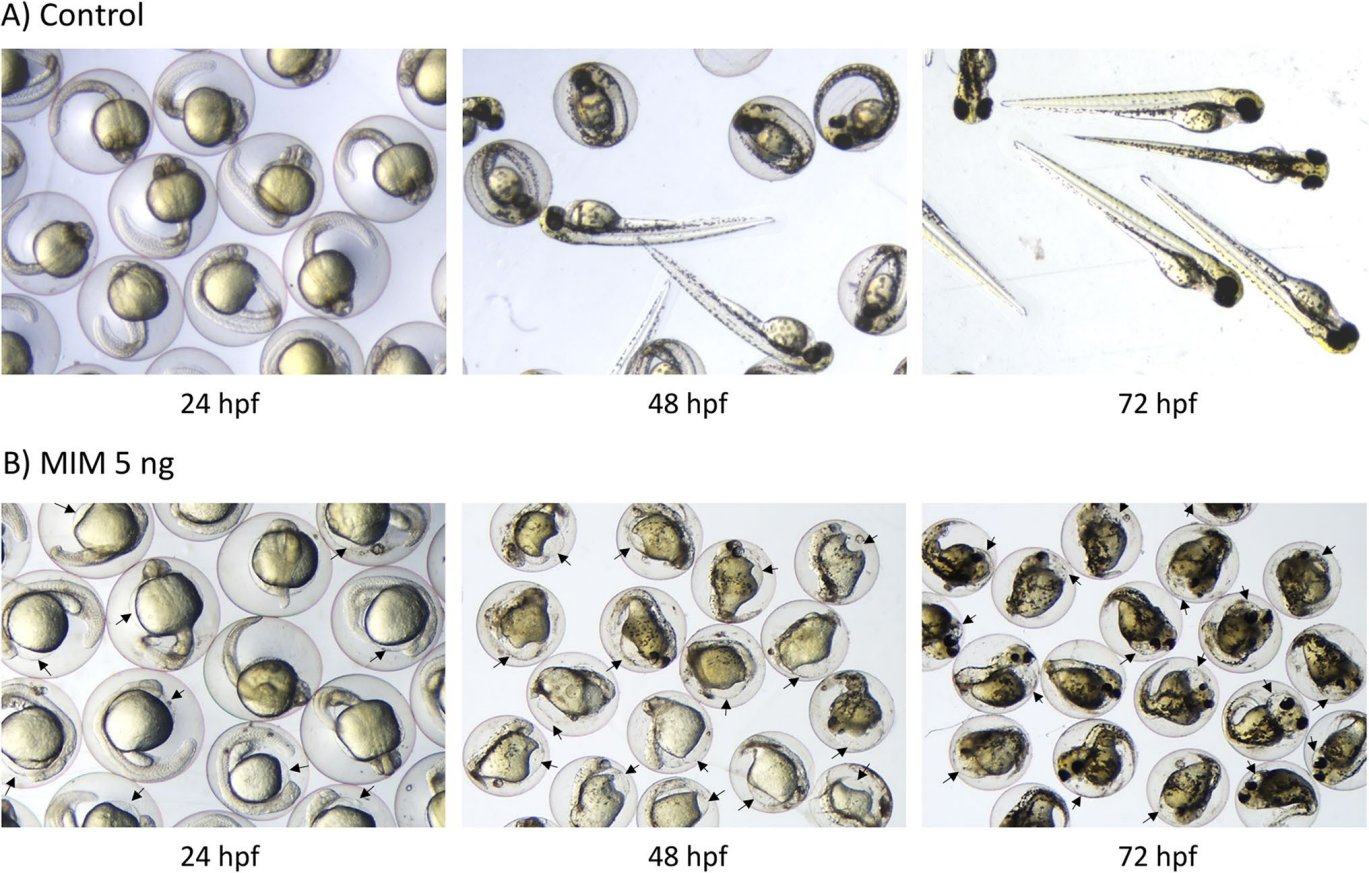
Development of zebrafish embryos and larvae throughout the experiment. **A** Normal development of control larvae and **B** developmental malformations in embryos and larvae after intra-yolk microinjections with MiR92b-3p at 5 ng/embryo (MIM 5 ng). Embryos from the exposed group exhibited a yolk void at 24 hpf that developed into pericardial edema (black arrows)

**Fig. 3:**
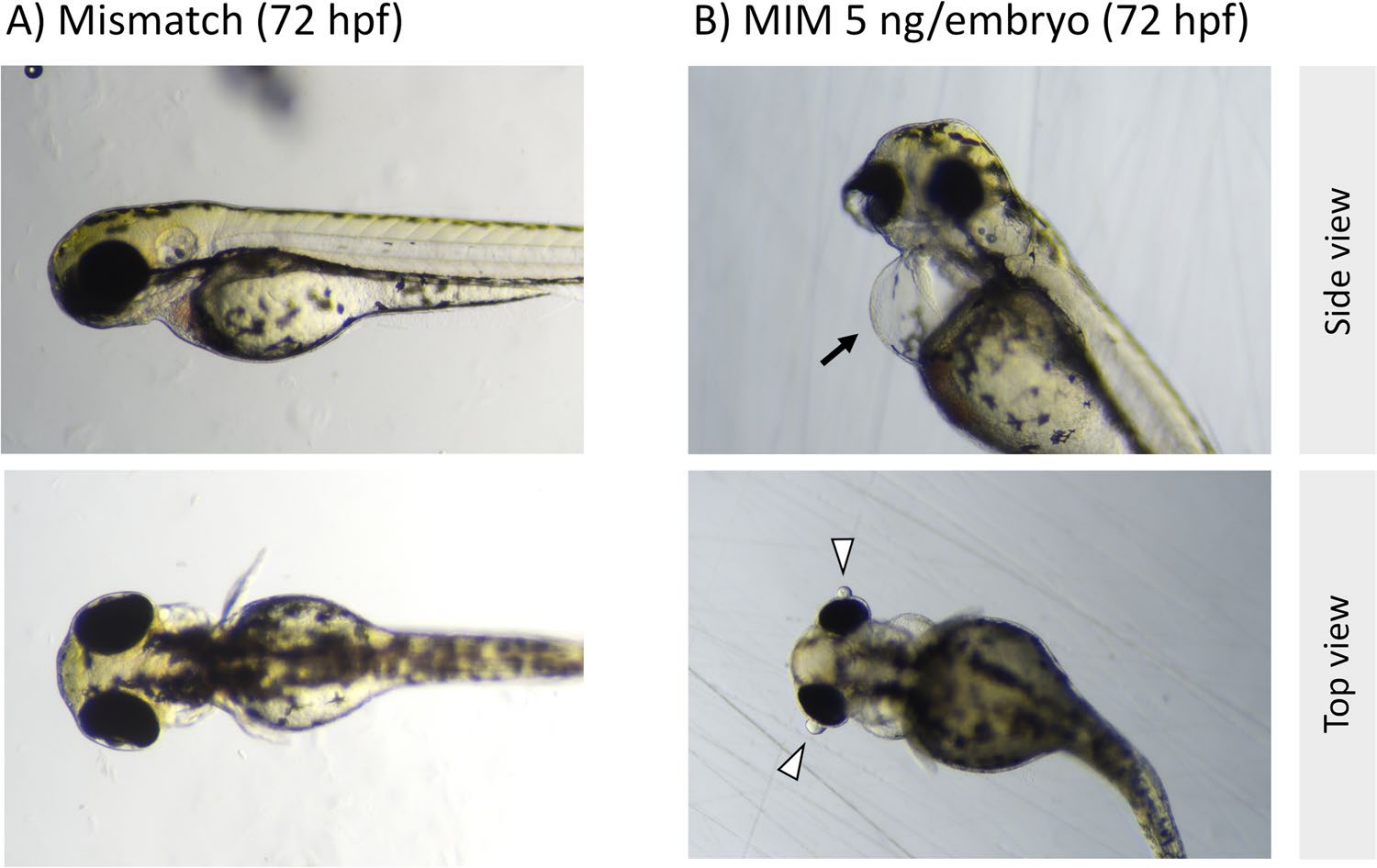
Representative phenotypes of zebrafish larvae 72 h after intra-yolk microinjections with **A** *C. elegans* MiR39-3p at 5 ng/embryo (mismatch) or **B** MiR92b-3p mimic at 5 ng/embryo (MIM 5 ng). In the mismatch group, zebrafish larvae had normal phenotypes, whereas the MIM-exposed larvae developed pericardial edema (black arrows) and had smaller eyes with abnormal morphology (white arrowheads)

Figures 2 and 3 show typical phenotypes of zebrafish embryos (at 24 and 48 hpf) and larvae (at 72 hpf) in the untreated (control) group and in the groups exposed to synthetic miRNA mimics (MIS or MIM). In the control group and the MIS-exposed group, the individuals had mostly normal phenotypes (Figs. 2A and 3A), but the MIM-treated zebrafish exhibited numerous developmental abnormalities throughout the experiment (Figs. 2B and 3B). Among the developmental defects commonly observed in the MIM-treated zebrafish were pericardial edema (black arrows) with abnormally thin, elongated heart chambers, and trunk and/or tail curvature (Fig. 3B). Additionally, the MIM-treated zebrafish developed smaller eyes with protruding corneas (white arrowheads). Importantly, this abnormal eye morphology was not observed in the malformed individuals in the other groups.

### Histological analysis of eyes

**Fig. 4:**
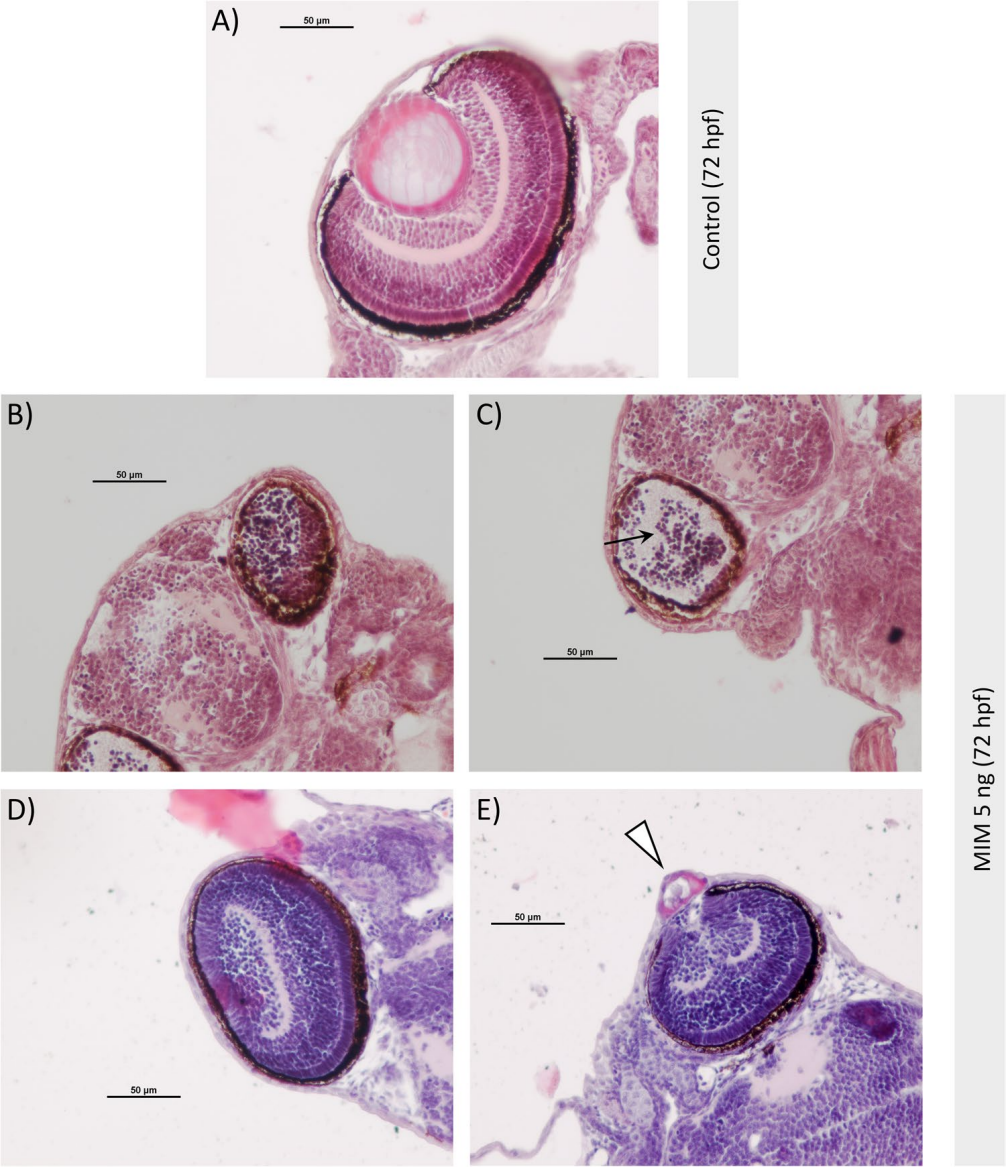
Morphology of eyes of zebrafish larvae at 72 hpf. **A** Normal eye structure of larvae from the control group and **B**–**E** morphological abnormalities of eyes in larvae microinjected with MiR92b-3p mimic at 5 ng/embryo (MIM 5 ng). The morphological changes in the exposed zebrafish usually manifested as smaller eye diameter, and as a lack of a lens and typical retina stratification (black arrow). In some zebrafish individuals, a vacuolized lens (or its remnants) was located in the delaminated cornea that was expanding outward (white arrowhead)

The histological structure of 72 hpf zebrafish sections was examined using light microscopy (Fig. 4). These examinations focused on the eyes because, after exposure to other substances, abnormal cornea protrusion is less commonly observed than pericardial edema or spine/trunk deformities (Haldi et al. 2012). Thus, cornea protrusion seemed to be more specific to the MIM exposure than the other abnormalities.

In the untreated (control) group, the eye structure was normal (Fig. 4A). Tissue stratification was normal, and the sclera, photoreceptor layer, inner nuclear layer, inner plexiform layer, ganglion cell layer, and cornea were visible. A spherical lens was located between the ganglion cell layer and the cornea, which consisted of a solid mass of fiber cells bounded by epithelium.

In the MIM-exposed zebrafish, two patterns of abnormalities were generally observed. First, in several larvae, the structure of the eye was grossly disturbed. The diameter of the eye was markedly reduced, the lens was absent, and normal tissue stratification was not present. Apparently normal sclera surrounded a cellular mass in which only partial stratification was visible (Fig. 4B). It was difficult to determine exactly which cellular layer was responsible for forming this cellular mass. Some of the eyes with a reduced diameter and without a lens were only loosely filled with an undetermined cellular mass, and a large empty space was present in these eyes (black arrow; Fig. 4C).

Second, in other MIM-exposed larvae, the eye diameter was also reduced, but the tissue was stratified like that in the control larvae. Importantly, pink eosin-stained remnants of the lens were visible in some specimens (Fig. 4D). In some other specimens, the lens was dislocated: a smaller, vacuolized lens was located in a delaminated cornea expanding outside the optic cup (white arrowhead; Fig. 4E). Although the stratification of these eyes was close to normal, it seems that cells of the ganglion cell layer tended to fill the space that normally would have been filled by the lens.

### Locomotor activity

**Fig. 5:**
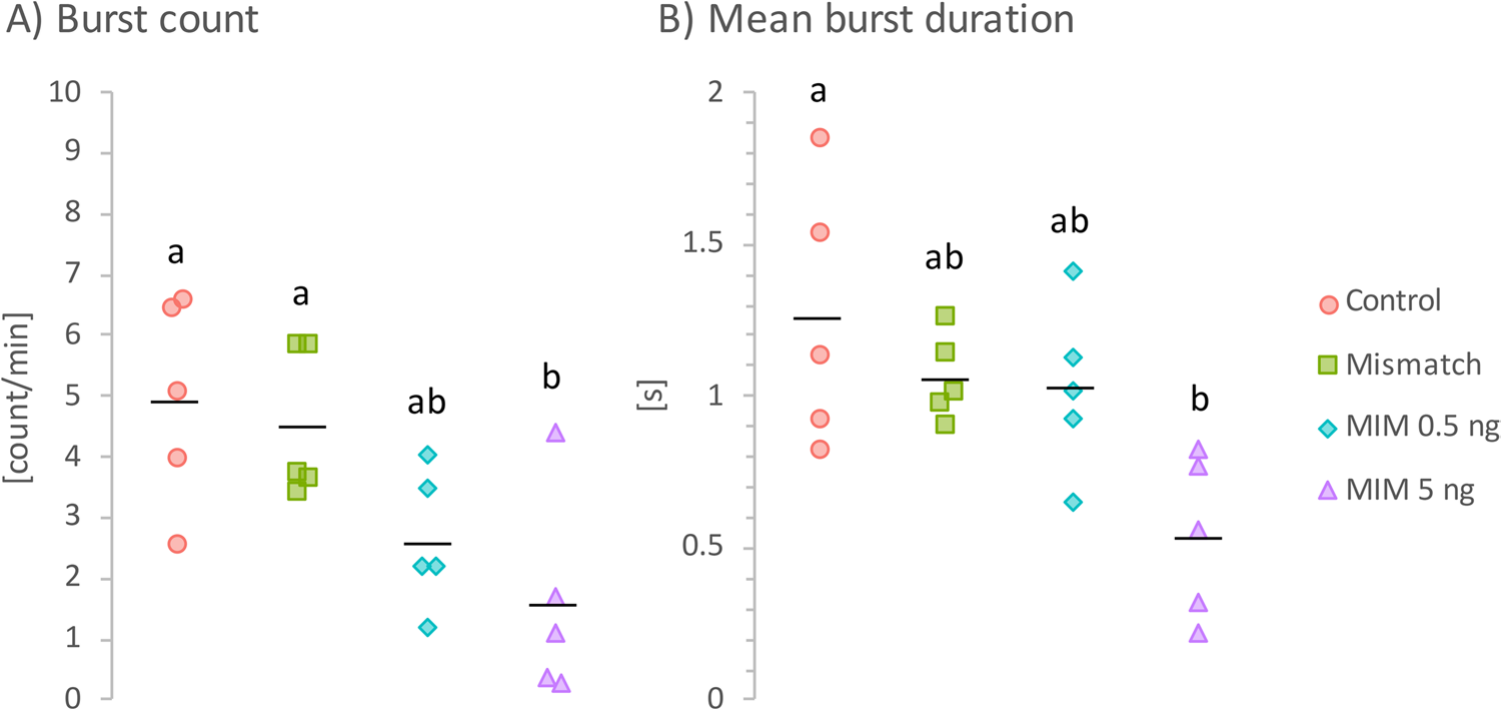
Locomotor activity of zebrafish embryos at 24 hpf. **A** Burst count and **B** mean burst duration of zebrafish that received intra-yolk microinjections of MiR92b-3p mimic at 0.5 ng/embryo (MIM 0.5 ng; cyan diamonds) or 5 ng/embryo (MIM 5 ng; violet triangles), those that received *C. elegans* MiR39-3p mimic at 5 ng/embryo (mismatch; green squares), or those that were not treated with microinjections (control; red circles). Individual points represent values from individual Petri dishes used for incubation (i.e., replicates; *n* = 5 per group). Black horizontal lines indicate group means, and different letters indicate statistically significant differences between group means (one-way independent ANOVA followed by Tukey’s HSD test; *p* < 0.05)

To examine whether the exposure to MIM affected embryonic movement, tail-coiling contractions (burst count and mean burst duration) of 24 hpf embryos were assessed (Fig. 5). In general, exposure to MIM decreased the frequency of embryo movement (Fig. 5A). In the untreated (control) group, the embryos moved inside their chorions at an average of 5 bursts/min. The burst count was 47% lower in the MIM 0.5 ng group (*p* = 0.09) and 68% lower in the MIM 5 ng (*p* = 0.01). In the MIS-treated group, although mean burst count was 8.7% lower than in control group, the difference was not significant (*p* = 1.0).

Additionally, the exposure to MIM also decreased the duration of bursts, but only the higher dose caused a substantial reduction (Fig. 5B). Compared to the control group, in which an average burst lasted 1.26 s, the duration was 57% shorter in the MIM 5 ng group (*p* = 0.008). In the rest of the groups (MIS and MIM 0.5), the average burst duration was also shorter (~ 17%) than in the control group, but the differences were not significant (*p* > 0.05).

### Distribution of the mimic in zebrafish after microinjection

**Fig. 6:**
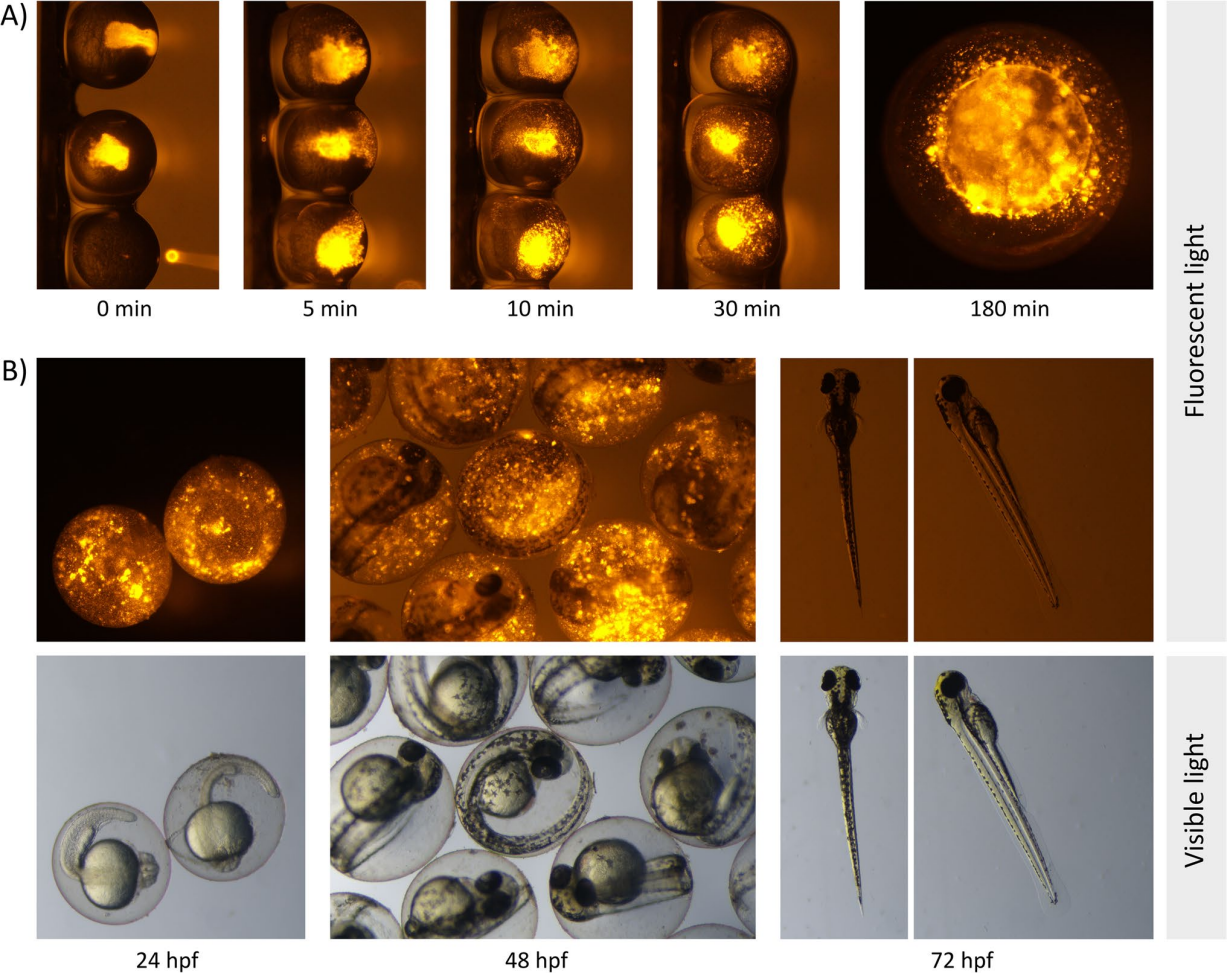
Successful delivery of MiR92b-3p into fertilized zebrafish eggs via intra-yolk microinjections. **A** Distribution of the MiR92b-3p mimic 3′-labeled with Alexa Fluor 555 in the yolks of the fertilized eggs up to 180 min after mimic injection at a dose of 1 ng/embryo (AF-MIM). **B** Localization of the fluorescent material in the zebrafish at 24, 48, and 72 hpf. Strong fluorescence was observed inside the eggs of the developing zebrafish embryos, but it was not observed in the hatched larvae at 72 hpf

To verify that the delivery of the mimic was successful, the distribution of a fluorescently labeled mimic (AF-MIM) in the microinjected embryos was examined (Fig. 6). Immediately after the injection, the orange fluorescent material was found inside the yolk sac; within the next 180 min, it spread throughout the sac. Starting 10 min after the injection, the material began to form lumps of various sizes and migrate from the yolk sac to the perivitelline space (Fig. 6A), spreading throughout the egg by 24 hpf. At 48 hpf, the fluorescent lumps seemed to be located in the perivitelline space rather than in the body of the embryos. However, the material was not observed inside hatched larvae at 72 hpf (Fig. 6B).

### Levels of MiR92(a)b-3p in hatched larvae

**Fig. 7:**
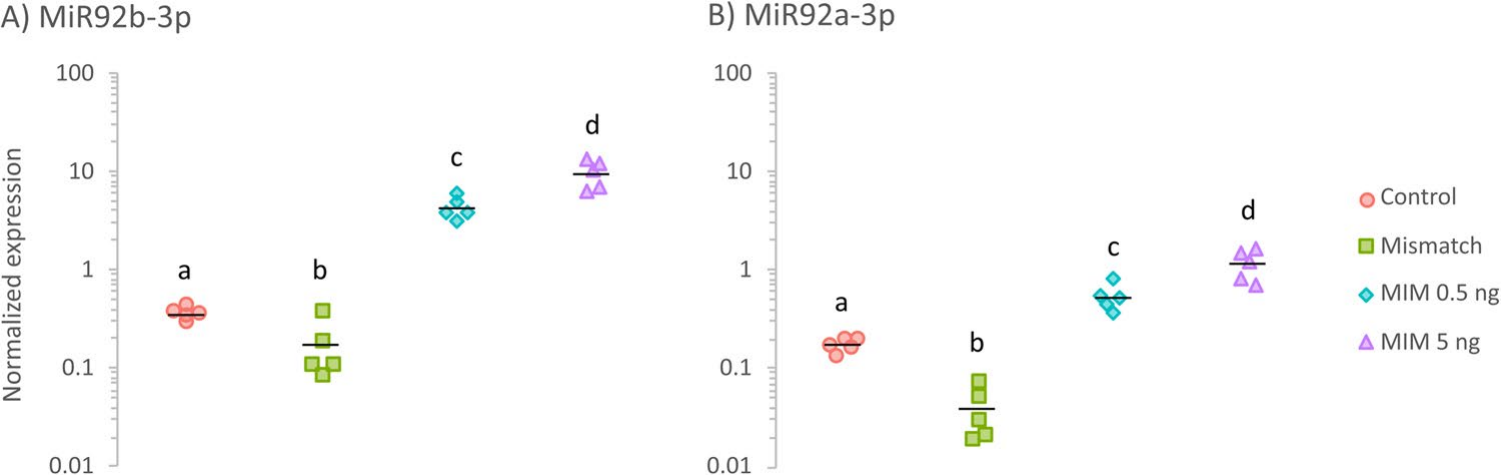
Relative expression of **A** MiR92b-3p and **B** MiR92a-3p in zebrafish larvae (at 72 hpf) that received intra-yolk microinjections of MiR92b-3p mimic at 0.5 ng/embryo (MIM 0.5 ng; cyan diamonds) or 5 ng/embryo (MIM 5 ng; violet triangles), those that received *C. elegans* MiR39-3p mimic at 5 ng/embryo (mismatch; green squares), or those that were not treated with microinjections (control; red circles). Individual points represent values from individual Petri dishes used for incubation (i.e., replicates; *n* = 5 per each group). Black horizontal lines indicate means, and different letters indicate statistically significant differences between group means (one-way independent ANOVA followed by Tukey’s HSD test; *p* < 0.05)

Since observations of the AF-MIM group did not clearly demonstrate the presence of the mimic in the bodies of the exposed zebrafish, the MiR92b-3p level in 72 hpf larvae from the control and MIM-treated groups was examined to confirm the efficacy of mimic-delivery (Fig. 7A). The intra-yolk microinjections with the mimic significantly increased the MiR92b-3p level in the zebrafish larvae (*p* < 0.001): relative to the untreated (control) group, there was a 12-fold increase in the MIM 0.5 ng group, and an over 25-fold increase in the MIM 5 ng group.

To check the specificity of miRNA-quantification with qPCR, MiR92a-3p was examined (Fig. 7B). Levels of MiR92a-3p in the MIM-treated larvae were also significantly higher than those in the control larvae (*p* < 0.001): relative to the control group, there was a threefold increase in the MIM 0.5 ng group and an almost sevenfold increase in the MIM 5 ng group. Importantly, however, the level of MiR92a-3p in each group was lower than that of MiR92b-3p.

Interestingly, the intra-yolk microinjections with *C. elegans* MiR39-3p at 5 ng/embryo (MIS) significantly decreased levels of both examined miRNAs in the zebrafish larvae (*p* < 0.001): relative to the control group, there was a twofold decrease of MiR92b-3p level and almost 4.5-fold decrease of MiR92a-3p level in the MIS group (Fig. 7).

### Expression level of mRNAs selected as potential targets for the mimic’s action

**Fig. 8:**
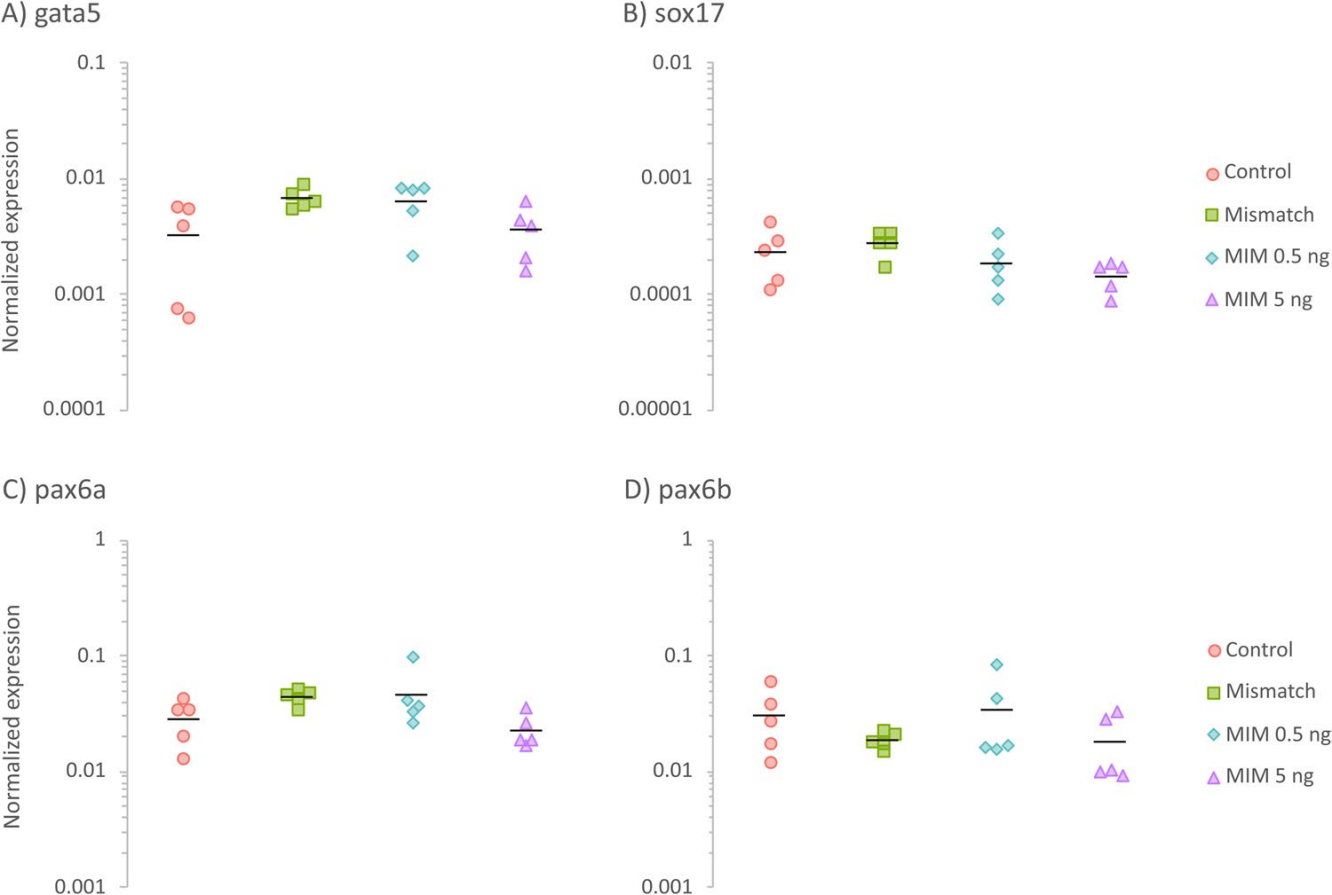
Relative expression of **A** *gata5*, **B** *sox17*, **C** *pax6a*, and **D** *pax6b* mRNA in zebrafish larvae (at 72 hpf) that received intra-yolk microinjections of MiR92b-3p mimic at 0.5 ng/embryo (MIM 0.5 ng; cyan diamonds) or 5 ng/embryo (MIM 5 ng; violet triangles), those that received *C. elegans* MiR39-3p mimic at 5 ng/embryo (mismatch; green squares), or those that were not treated with microinjections (control; red circles). Individual points represent values collected from individual Petri dishes used for incubation (i.e., replicates; *n* = 5 per each group). Black horizontal lines indicate means

To gain information on possible regulatory mechanisms responsible for the morphological abnormalities in the eyes of the exposed zebrafish, the mRNA expression of genes that the literature indicated or suggested as targets for MiR92b-3p was screened (Fig. 8). All the differences in the mRNA levels of the selected genes were not statistically significant (*p* > 0.05); however, the mRNA levels were always lower in the MIM 5 ng group than in the MIM 0.5 ng group.

## Discussion

### Effects of mimic microinjections on miRNA levels in zebrafish

This study assessed the role of MiR92b-3p in the embryonic development of zebrafish by administering a synthetic miRNA analogue: mirVana MiR92b-3p mimic (MIM). Microinjections with this mimic can be used to modulate miRNA levels in the bodies of zebrafish, as shown by the spread of the fluorescently labeled mimic inside the entire egg by 24 hpf (Fig. 6) and the elevated levels of MiR92b-3p in the exposed zebrafish at 72 hpf (Fig. 7A).

It was also found that levels of another miRNA, MiR92a-3p, were increased in both MIM-exposed groups (Fig. 7B), although this increase was much smaller than the increase in MiR92b-3p levels. MiR92a-3p belongs to the same precursor family as MiR92b-3p. These two miRNA species share the same seed region, and their mature sequences differ by only a few nucleotides (MIMAT0001808 vs. MIMAT0001809; miRBase). Thus, the increased MiR92a-3p level after MIM exposure seems likely to be the result of a lack of complete specificity on the part of the stem-loop qPCR assay, which probably could not completely distinguish between the two short and highly similar mature miRNA sequences (Schamberger and Orbán 2014; Magee et al. 2017).

However, the reasons for the probable lack of complete assay specificity also suggest that these two homologous miRNAs share molecular targets and biological functions. Although Brodersen and Voinnet (2009) showed that miRNA secondary structure may be pivotal for potential miRNA-mRNA interactions, Jonas and Izaurralde (2015) found that, in most animal-cell interactions, the miRNA and target site sequence do not need to be 100% complementary if the seed region matches. Similarly, Wang (2014) concluded that, of all the factors they examined, the seed region’s complementarity with miRNA is crucial for triggering any effect.

### MiR92b-3p mimic impairs embryonic development, which leads to death or problems with hatching

Both doses of MIM increased mortality and the rate of malformation occurrence in the zebrafish embryos. Moreover, the hatching rate of the exposed groups was markedly lower than that of the control or MIS-exposed groups (Fig. 1). Importantly, the developmental defects that were observed in the MIM-exposed groups were severe (pericardial edema and abnormal heart morphology), and some of them involved the musculoskeletal system (trunk/tail curvature; Figs. 2 and 3). These results suggest that, during zebrafish embryogenesis, MiR92b-3p overexpression may impair their development, leading to death or problems with hatching.

The present findings are consistent with those of other studies, which demonstrate that MiR92a and/or MiR92b may play controlling roles in organogenesis and overall body patterning in fish (Kloosterman et al. 2006; Li et al. 2011, 2020; Ning et al. 2013). For example, microinjections of fertilized zebrafish eggs with a mixture of MiR92a and MiR92b (MiR92) selectively impaired endoderm formation, leading to developmental heart defects during early embryonic development; the MiR92-injected embryos displayed a failure of heart fusion or an inability of the heart tube to undergo normal looping (Li et al. 2011). Similarly, in a study on Japanese flounder, injection with MiR92 induced spinal deformities in the exposed embryos (Li et al. 2020). Taken together, the findings of the present study and others suggest that MiR92b-3p could be an important regulatory factor in fish embryogenesis.

### MiR92b-3p mimic decreases the movement of zebrafish embryos

The frequency of embryo movements in the control group of this study was consistent with counts previously reported for normal zebrafish embryos (Basnet et al. 2017; Cheng et al. 2017; Liu et al. 2018). However, MIM exposure not only decreased the frequency of embryo movements inside the chorion (burst count; Fig. 5A), but also shortened average movement time (average burst duration; Fig. 5B). This movement decrease was probably at least partially due to the developmental abnormalities in the MIM-treated groups, which likely made it physically difficult to move their tails and/or may have affected nervous system function (e.g., signal transmission in the spinal cord). The decreased tail movement in the MIM-treated groups may also have been a cause of the reduced hatching rate in these groups, as these embryos may not have been able to move with enough strength or often enough to break through the chorion.

### MiR92b-3p mimic affected retinal differentiation and lens formation during zebrafish embryogenesis

Evidence for these effects of MiR92b-3p mimic is provided by several observations (Figs. 3 and 4). First, the eyes of the MIM-exposed zebrafish exhibited morphological abnormalities and were generally smaller than those of the control fish. Second, in the eyes of several MIM-treated larvae, the lens was absent, and the retina lacked normal tissue stratification (Fig. 4B and C). Finally, in other individuals, the lens was present, and the retinal tissue was stratified, but the lens was much smaller, vacuolized, and dislocated outside the optic cup (Fig. 4D and E). Importantly, because these malformations were not observed in the MIS-treated group, the possibility that the eye defects were caused by a non-specific action of the synthetic miRNA can be excluded.

To discuss the pathological processes that could have led to the observed abnormalities in eye morphology, it is first necessary to review the process of normal ocular development in zebrafish (Kimmel et al. 1995; Dahm et al. 2007; Richardson et al. 2017). At around 28 hpf, zebrafish eyes consist of an optic cup composed of two retinal layers. While the optic cup is developing, the lens develops from the lens placode, which originates from the surface ectoderm cells overlying the optic cup. The lens placode progressively delaminates from the surface ectoderm, resulting in the formation of a solid lens mass inside the optic cup. This lens mass then detaches from the ectoderm and differentiates into primary lens fiber cells and a surrounding layer of anterior epithelium. In the present study, the small, vacuolized, and dislocated lenses (or a lack of lenses) in the eyes of the MIM-exposed zebrafish suggest that MiR92b-3p could have inhibited (or blocked) the formation and differentiation of the solid lens mass from the lens placode. Since the lens develops concomitantly with the optic cup (Dahm et al. 2007; Richardson et al. 2017), impaired formation of the solid lens mass (or a lack of lens mass formation) could have prevented correct invagination of the optic cup, thus leading to decreased eye diameter in the exposed zebrafish.

To the best of our knowledge, this is the first report on the effects of MiR92b-3p mimic on ocular development. However, there are a few reports of zebrafish mutants that display ocular phenotypes similar to those of the MIM-treated zebrafish in the present study (Nadauld et al. 2006; Moosajee et al. 2008; Kleinjan et al. 2008; Takamiya et al. 2015; Taler et al. 2020). For example, the eyes of *apc*-deficient mutants exhibited protruding lenses and a lack of retinal organization caused by impaired differentiation of retinal progenitor cells (Nadauld et al. 2006). These ocular defects were attributed to the dual role of *apc* in eye morphogenesis: (i) regulation of the Wnt/β-catenin cascade during lens development and (ii) control of retinoic acid synthesis, which is necessary for proper development and differentiation of the retina (Nadauld et al. 2006). Mutation of another gene, *plod3*, resulted in malformed embryos without lenses or with dislocated lenses, among other deformities (Taler et al. 2020). *plod3* codes for an enzyme required for modifying and secreting collagens; this suggests that the abnormal lens morphology was caused by impaired synthesis of collagen IV in the lens capsule, which supports and maintains the shape and correct position of the lens (Taler et al. 2020). Together, the results of the present study and previous ones provide insight into the complexity of the genetic networks and cellular mechanisms involved in the spatiotemporal regulation of eye morphogenesis.

### Mimic exposure did not clearly affect mRNA expression of putative MiR92b-3p targets

Based on literature indicating their role in ocular development or their likely ability to interact with MiR92b-3p, the expression of several genes was examined (Fig. 8). Interestingly, although the changes in the mRNA levels of these genes were not statistically significant, their expression was always lower in the MIM 5 ng group than in the MIM 0.5 ng group. According to the American Statistical Association’s Statement on *p* values (Wasserstein and Lazar 2016), articles by statistical experts (including Cumming 2014; Greenland et al. 2016; Amrhein et al. 2017), and standard statistical textbooks (including Kirkwood and Sterne 2003; Field et al. 2012; Motulsky 2018), statistically non-significant results are correctly interpreted as inconclusive, including those with large *p* values. Therefore, the results of the present study are not inconsistent with those of previous research, which indicated that the role of MiR92 in endoderm formation and body patterning may arise from its direct molecular interference with *sox17* and *gata5* expression. For example, although they are statistically inconclusive, the present results suggest that MIM-injection may reduce *sox17* mRNA expression (Fig. 8E). Similarly, Li et al. (2011) observed that injection of MiR92 into zebrafish embryos significantly decreased the number of *sox17*-expressing cells. As for *gata5*, the present results produced wide 95% confidence intervals, which include potential differences like those observed by Li et al. (2020), who reported that exposure to MiR92 decreased expression of both *gata5* and *sox17* in Japanese flounder embryos at early developmental stages. Thus, in this context, the present *sox17* results are suggestive, and thanks to the development of meta-analytical statistical techniques (e.g., Cumming 2014), the results of the present study can be combined with those of other studies to estimate the effects of MiR92-injection on gene expression more precisely.

## Conclusion

The present study demonstrates that exposure of zebrafish embryos to a synthetic MiR92b-3p mimic impairs their development, leading to increased occurrence of malformations, decreased locomotor activity, problems with hatching, and increased mortality. Importantly, the MiR92b-3p mimic affected retinal differentiation and lens formation during zebrafish embryogenesis, which suggests that MiR92b-3p could be an important factor in the regulation of fish embryogenesis and ocular development. Further research is needed to identify the MiR92b-3p–regulated cell pathways involved in the pathological course of embryogenesis observed in the zebrafish.

## Supplementary Information

Below is the link to the electronic supplementary material.

Supplementary file1 (PDF 431 KB)
