# Supplementary material for: MiR92b-3p synthetic analogue impairs zebrafish embryonic development, leading to ocular defects, decreased movement and hatching rate, and increased mortality
Source: J Appl Genet. 2022 Oct 24;64(1):145–57. doi: 10.1007/s13353-022-00732-w (PMC9837005; doi:10.1007/s13353-022-00732-w)
Supplement: Supplementary file 1 — Supplementary file1 (PDF 431 KB) [file 13353_2022_732_MOESM1_ESM.pdf]

Supplement 1

Table 1. Details of the qPCR primers used in this study to amplify target and reference sequences.

Name	Sequence [5'→3']	Amplicon length [bp]	Final primer concentration [µM each]	Reference
		mRNAs		
gata5	F: ctgcctgtgtcagaaaacgc<br>R: aactgtgtcgatgcctgtgt	107	0.5	NM_131235
sox17	F: gcatccgaaggccaatgaac<br>R: gctttccatgacttaccaagca	124	0.5	NM_131287
pax6a	F: ggaacgggtttattgagggaa<br>R: acagcctttgtatcctcgct	147	0.5	NM_131304
pax6b	F: accgcttccacaggactcatc<br>R: aaagggcgtagctcttttctttac	119	0.5	NM_131641
actb1	F: tgagcaggagatgggaacc<br>R: caacggaaacgctcattgc	102	0.5	Leach et al. 2020
eef1a1l1	F: gagaagttcgagaaggaagc<br>R: cgtagtatttgctggtctcg	142	0.5	Maculay et al. 2016
ubc	F: aagagactcccatacaccgc<br>R: attctcaatggtgtcgctgg	128	0.5	NM_001077804
		ncRNAs		
MiR92a-3p	F: acactccagctgggtattgcacttgtccc<br>R: ctcacagtacgttggtatccttgtg	~100	1	F: MIMAT0001808<br>R: Biggar et al. 2014
MiR92b-3p	F: attattgcactcgtcccggcct<br>R: ctcacagtacgttggtatccttgtg	~100	1	F: MIMAT0001809<br>R: Biggar et al. 2014
rnu6	F: tcgcttcggcagcacata<br>R: ctcacagtacgttggtatccttgtg	~100	1	F: Zhuang et al. 2014<br>R: Biggar et al. 2014

Table 2. Efficiency and specificity of qPCR indicated by details of the standard curve, melting curve analysis (dissociation), and agarose gel electrophoresis of the amplicons (part 1).

Name	*gata5*	*sox17*
Specificity	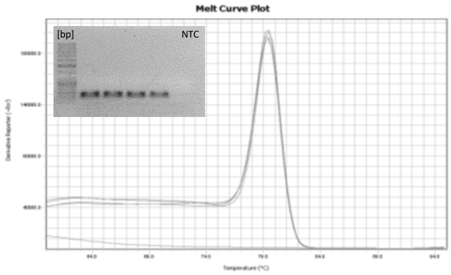	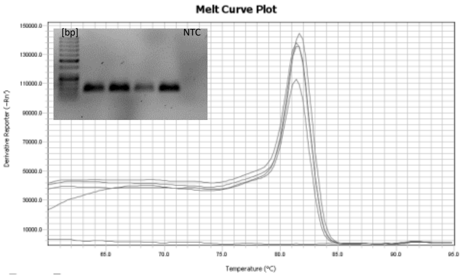
Tm [°C]	79.2	81.5
Standard curve	y=35.646x–3.362 R^2=0.991	y=42.253x–3.781 R^2=0.988
Cq [mean ±S.D.]	29.29 ±2.45	33.7 ±1.94
M-value	0.851	0.627
Name	*pax6a*	*pax6b*
Specificity	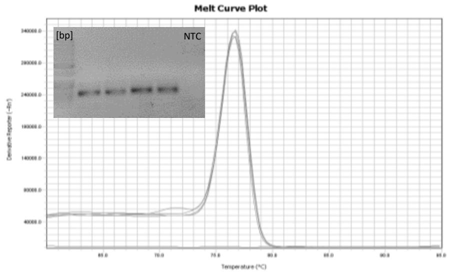	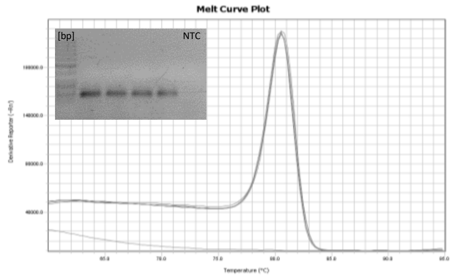
Tm [°C]	76.6	80.5
Standard curve	y=34.221x–3.649 R^2=0.992	y=34.592x–3.475 R^2=0.998
Cq [mean ±S.D.]	26.32 ±1.97	26.92 ±1.61
M-value	0.547	0.716
Name	*actb1*	*eef1a1l1*
Specificity	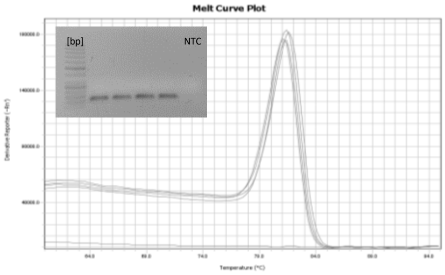	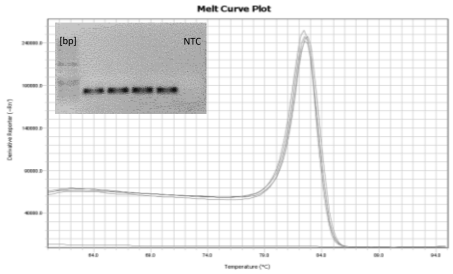
Tm [°C]	81.2	82.4
Standard curve	y=26.764x–3.386 R^2=0.999	y=25.684x–3.458 R^2=0.998
Cq [mean ±S.D.]	19.76 ±1.94	18.56 ±2.06
M-value	0.427	0.460
Name	*ubc*	
Specificity	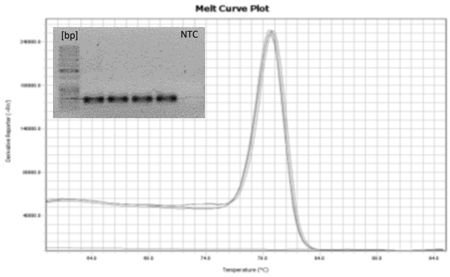	
Tm [°C]	79.5	
Standard curve	y=29.01x–3.45 R^2=0.998	
Cq [mean ±S.D.]	21.36 ±1.68	
M-value	0.427	

Table 3. Efficiency and specificity of qPCR indicated by details of the standard curve, melting curve analysis (dissociation), and agarose gel electrophoresis of the amplicons (part 2).

Name	MiR92a-3p	MiR92b-3p
Specificity	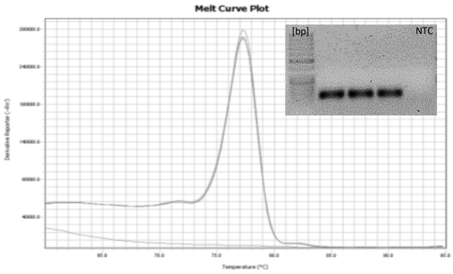	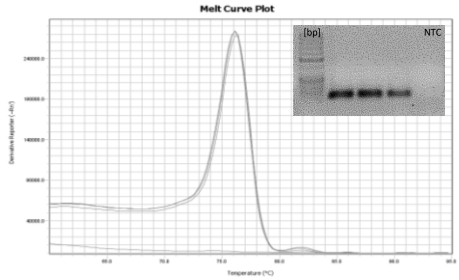
Tm [°C]	77.2	76.2
Standard curve	y=31.225x–3.318 R^2=0.994	y=28.395x–3.351 R^2=0.999
Cq [mean ±S.D.]	24.85 ±2.26	22.63 ±2.77
M-value	1.673	2.061
Name	rnu6	
Specificity	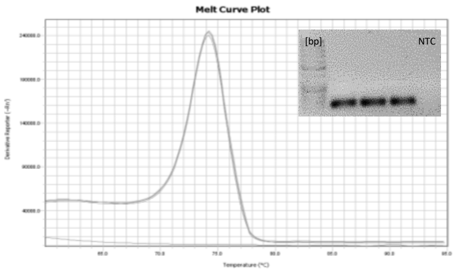	
Tm [°C]	74.4	
Standard curve	y=28.473x–3.303 R^2=0.999	
Cq [mean ±S.D.]	22.82 ±0.61	
M-value	1.185	
